# Cost-effectiveness analysis of using the *TBX6*-associated congenital scoliosis risk score (TACScore) in genetic diagnosis of congenital scoliosis

**DOI:** 10.1186/s13023-020-01537-y

**Published:** 2020-09-15

**Authors:** Zefu Chen, Zihui Yan, Chenxi Yu, Jiaqi Liu, Yanbin Zhang, Sen Zhao, Jiachen Lin, Yuanqiang Zhang, Lianlei Wang, Mao Lin, Yingzhao Huang, Xiaoxin Li, Yuchen Niu, Shengru Wang, Zhihong Wu, DISCO (Deciphering disorders Involving Scoliosis and COmorbidities) study group, Guixing Qiu, Zhihong Wu, Jianguo Zhang, Nan Wu, Lan Zhu, Shengru Wang, Na Chen, Jiaqi Liu, Sen Liu, Yuzhi Zuo, Gang Liu, Yuanqiang Zhang, Chenxi Yu, Sen Zhao, Lianlei Wang, Yanxue Zhao, Weisheng Chen, Zihui Yan, Xinzhuang Yang, Hengqiang Zhao, Yuchen Niu, Jingdan Chen, Xiaoxin Li, Huizi Wang, Zhi Zhao, Yiran Cui, Zixin Zhang, Zefu Chen, Bowen Liu, Xi Cheng, Mao Lin, Jiachen Lin, Huakang Du, Yaqi Li, Yi You, Guixing Qiu, Terry Jianguo Zhang, Nan Wu

**Affiliations:** 1 Department of Orthopedic Surgery, Peking Union Medical College Hospital, Peking Union Medical College and Chinese Academy of Medical Sciences, Beijing, 100730 China; 2 Beijing Key Laboratory for Genetic Research of Skeletal Deformity, Beijing, 100730 China; 3 Graduate School of Peking Union Medical College, Beijing, 100005 China; 4 Department of Breast Surgical Oncology, National Cancer Center/Cancer Hospital, Chinese Academy of Medical Sciences and Peking Union Medical College, Beijing, China; 5 Medical Research Center, Peking Union Medical College Hospital, Peking Union Medical College and Chinese Academy of Medical Sciences, Beijing, China; 6 Key Laboratory of Big Data for Spinal Deformities, Chinese Academy of Medical Sciences, Beijing, 100730 China

**Keywords:** *TBX6*-associated congenital scoliosis, *TBX6*-associated congenital scoliosis risk score, Whole-exome sequencing, Molecular diagnosis, Cost-effectiveness analysis

## Abstract

**Background:**

We previously reported a novel clinically distinguishable subtype of congenital scoliosis (CS), namely, *TBX6*-associated congenital scoliosis (TACS). We further developed the *TBX6*-associated CS risk score (TACScore), a multivariate phenotype-based model to predict TACS according to the patient’s clinical manifestations. In this study, we aimed to evaluate whether using the TACScore as a screening method prior to performing whole-exome sequencing (WES) is more cost-effective than using WES as the first-line genetic test for CS.

**Methods:**

We retrospectively collected the molecular data of 416 CS patients in the Deciphering disorders Involving Scoliosis and COmorbidities (DISCO) study. A decision tree was constructed to estimate the cost and the diagnostic time required for the two alternative strategies (TACScore versus WES). Bootstrapping simulations and sensitivity analyses were performed to examine the distributions and robustness of the estimates. The economic evaluation considered both the health care payer and the personal budget perspectives.

**Results:**

From the health care payer perspective, the strategy of using the TACScore as the primary screening method resulted in an average cost of $1074.2 (95%CI: $1044.8 to $1103.5) and an average diagnostic duration of 38.7d (95%CI: 37.8d to 39.6d) to obtain a molecular diagnosis for each patient. In contrast, the corresponding values were $1169.6 (95%CI: $1166.9 to $1172.2) and 41.4d (95%CI: 41.1d to 41.7d) taking WES as the first-line test (*P* < 0.001). From the personal budget perspective, patients who were predicted to be positive by the TACScore received a result with an average cost of $715.1 (95%CI: $594.5 to $835.7) and an average diagnostic duration of 30.4d (95%CI: 26.3d to 34.6d). Comparatively, the strategy of WES as the first-line test was estimated to have significantly longer diagnostic time with an average of 44.0d (95%CI: 43.2d to 44.9d), and more expensive with an average of $1193.4 (95%CI: $1185.5 to $1201.3) (*P* < 0.001). In 100% of the bootstrapping simulations, the TACScore strategy was significantly less costly and more time-saving than WES. The sensitivity analyses revealed that the TACScore strategy remained cost-effective even when the cost per WES decreased to $8.8.

**Conclusions:**

This retrospective study provides clinicians with economic evidence to integrate the TACScore into clinical practice. The TACScore can be considered a cost-effective tool when it serves as a screening test prior to performing WES.

## Background

Congenital scoliosis (CS), a relatively rare disease with a prevalence of approximately 0.5–1 per 1000 live births, is a kind of spinal curvature that results from vertebral malformations, including formation failure, segmentation failure, or a combination of the two [1, 2]. Genetic factors, vitamin deficiency, chemicals and environmental factors are assumed to independently or jointly contribute to CS [3, 4].

To date, with multiple clinical genetic measurements, we are able to molecularly classify CS according to their genetic etiologies [5]. We recently reported a novel clinically distinguishable subtype of CS, namely, *TBX6*-associated congenital scoliosis (TACS). Briefly, TACS is caused by *TBX6* loss-of-function variations compound with the common hypomorphic single nucleotide polymorphisms (defined by rs2289292-rs3809624-rs3809627), and is characterized by hemivertebrae or butterfly vertebrae in the lower half of the spine [6–9]. Compared with non-TACS patients, TACS patients demonstrate simpler rib anomalies and fewer intraspinal malformations. Based on the specific phenotype-genotype relationship, we developed the *TBX6*-associated CS risk score (TACScore), a multivariate phenotype-based model to predict TACS according to the patient’s clinical manifestations [7]. Specifically, the TACScore integrates the following variables: 1) segmented hemivertebrae or segmented butterfly vertebrae involving the lower half of the spine (T8-S5) (0 or 3 points); 2) the number of vertebral malformations (− 4 to 0 points); 3) the presence of intraspinal defects (− 3 or 0 points); and 4) the types of rib defect (− 1 to 1 points) (Table S1). The cutoff point of the TACScore was selected as 3 to achieve a high Youden index, with a sensitivity, specificity, and accuracy of 93.9%, 90.9% and 91.2% respectively [7].

Currently, whole-exome sequencing (WES) is recommended in clinical practice for patients suspected of Mendelian diseases, and is supposed to be implemented at the early stage of diagnosis to improve the cost-effectiveness [10]. However, compared with single-gene or a subset of genes (i.e. panel) test, WES is sometimes still considered costly and time-consuming for patients [11]. In this study, we aimed to compare whether applying the TACScore as a screening test prior to performing WES is more cost-effective than WES as the first-line test in molecularly diagnosing CS.

## Results

**Fig. 1:**
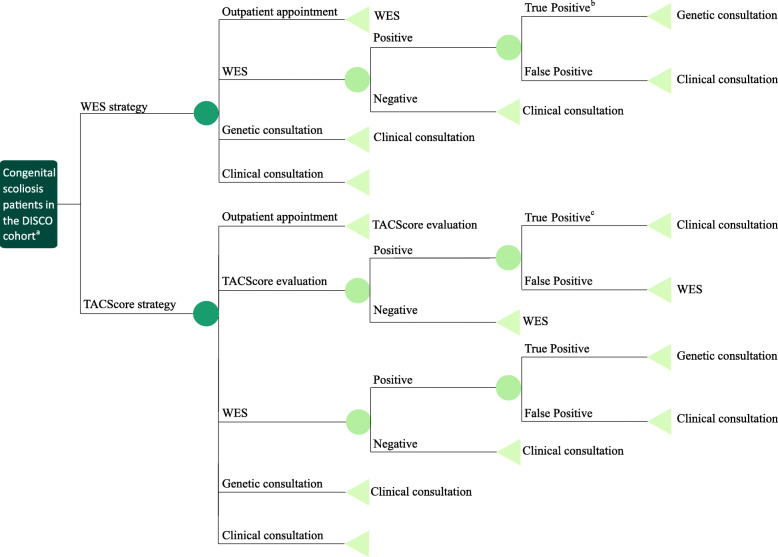
Decision tree of the WES strategy versus the TACScore strategy. In model 1, WES serves as the first-line test. In model 2, the TACScore serves as a screening test and WES is administered when the TACScore indicates a negative outcome. a. CS patients from the DISCO (Decipher of disorder Involving Scoliosis and COmorbidities) cohort. b. The SNVs and CNVs are called from the WES data. The SNVs are verified by Sanger sequencing, and the CNVs are confirmed by ddPCR. c. The true positive of the TACScore is confirmed by the *TBX6* monogenic test, which consists of Sanger sequencing of all exons and the approximately 1-kb upstream region of the *TBX6* gene, and the ddPCR detection of the 16p11.2 deletions. Abbreviations: WES, whole-exome sequencing; TACScore, *TBX6*-associated congenital scoliosis risk score; CS, congenital scoliosis; SNV, single-nucleotide variant; CNV, copy number variation; ddPCR, droplet digital polymerase chain reaction

We retrospectively collected the molecular data of 416 patients from the Deciphering disorders Involving Scoliosis and COmorbidities (DISCO) study. We designed a decision tree (Fig.1) and performed a cost-effectiveness analysis for all participants. The cost-effectiveness analysis focused on the costs and diagnostic times along the diagnostic trajectory: outpatient appointment, DNA extraction and shipping, WES, Sanger sequencing, droplet digital polymerase chain reaction (ddPCR), genetic consultations and clinical consultations (Table 1).

**Table 1:** The costs and diagnostic times required for investigations

Variables	Cost (USD)	Time (d)
Outpatient appointment	16.1	1
DNA extraction and shipping	29.9	1
Whole-exome sequencing	1094.8	37
Sanger sequencing	23.7	7
ddPCR	32.2	7
Genetic consultation	16.1	1
Clinical consultation	16.1	1

*Abbreviations: ddPCR* droplet digital polymerase chain reaction

### Cost-effectiveness analysis from the health care payer perspective

In counterfactual model 1 (the WES strategy), WES diagnosed a total of 73/416 (17.5%) patients, 42 of whom were diagnosed by *TBX6* variations (thus TACS) and 31 of whom were diagnosed by other genes [12]. The total cost of 416 patients was $486,545.4, and the total diagnostic time was 17,224d. The average cost per patient was $1169.6 (95% CI: $1166.9 to $1172.2). The average diagnostic time per patient was 41.4d (95% CI: 41.1d to 41.7d).

In counterfactual model 2 (the TACScore strategy), 83/416 (20.0%) patients were predicted to be positive by the TACScore (TACScore≥3) and 42 were diagnosed with TACS by the *TBX6* monogenic test (including Sanger sequencing and ddPCR). Subsequently, a total of 374 patients underwent WES, including 31 patients who were further diagnosed by other genes and 343 patients who received uninformative WES reports. The average cost per patient was reduced to $1074.2 (95% CI: $1044.8 to $1103.5), and the average diagnostic time per patient decreased to 38.7d (95% CI: 37.8d to 39.6d).

Compared with using WES as the first-line test (counterfactual model 1), using the TACScore as a primary screening prior to performing WES (counterfactual model 2) saved $95.4 (95% CI: $66.0 to $124.9, *P* < 0.001) and 2.7d (95% CI: 1.7d to 3.7d, P < 0.001) (Table 2). The TACScore strategy and the WES strategy shared an identical diagnostic rate (73/416, 17.5%). In this study, the incremental cost-effectiveness ratio (ICER) was not reported because the TACScore strategy is not only less costly but also more time-saving than the WES strategy.

**Table 2:** Cost-effectiveness of the TACScore strategy versus the WES strategy from the health care payer perspective

	Model 1: WES as a first-line test (USD)	Model 2: TACScore as a screening test (USD)
Assessments		
Outpatient appointment	6697.6	6697.6
Genetic consultation	1175.3	563.5
Clinical consultation	6697.6	6697.6
*TBX6* Monogenic test		
Sanger sequencing	1730.1	2796.6
ddPCR	2350.6	3799.6
Next generation sequencing		
WES	455436.8	413834.4
Other		
DNA extraction and shipping	12457.5	12457.5
Total cost	486545.4	446846.8
Total diagnostic time (d)	17224	16095
Total number of patients	416	416
Number of patients underwent WES	416	378
Total number of diagnoses	73	73
Average cost per patient (95% CI)	1169.6 (1166.9, 1172.2)	1074.2 (1044.8, 1103.5)
Average cost difference (95% CI)	–	−95.4 (−124.9, −66.0)
Average diagnostic time per patient (95% CI) (d)	41.4 (41.1, 41.7)	38.7 (37.8, 39.6)
Average diagnostic time difference (95% CI) (d)	–	−2.7 (−3.7, −1.7)

*Abbreviations: WES* whole-exome sequencing, *TACScore TBX6*-associated congenital scoliosis risk score, *ddPCR* droplet digital polymerase chain reaction

### Cost-effectiveness from the personal budget perspective

Regarding the patients who were predicted to be positive by the TACScore, in counterfactual model 1 (the WES strategy), WES diagnosed a total of 42/83 (17.5%) patients, 38 of whom were diagnosed by *TBX6* variations (thus TACS) and 4 of whom were diagnosed by other genes. The total cost of 83 patients was 99,050.5$, and the total diagnostic time was 3656d. The average cost per patient was $1193.4 (95% CI: $1185.5 to $1201.3). The average diagnostic time per patient was 44.0d (95% CI: 43.1d to 44.9d).

In counterfactual model 2 (the TACScore strategy), 38 were diagnosed with TACS by the *TBX6* monogenic test (including Sanger sequencing and ddPCR). Subsequently, a total of 45 patients underwent WES, including 4 patients who were further diagnosed by other genes and 41 patients who received uninformative WES reports. The total cost of 83 patients was 59,351.8$, and the total diagnostic time was 2527d. The average cost per patient was $715.1 (95% CI: $594.5 to $835.7) and the average diagnostic time per patient was 30.4d (95% CI: 26.3d to 34.6d) .

Compared with using WES as the first-line test (counterfactual model 1), using the TACScore as a primary screening prior to performing WES (counterfactual model 2) saved $478.3 (95% CI: $357.5 to $599.1, *P* < 0.001) and 13.6d (95% CI: 9.4d to 17.8d, P < 0.001) (Table 3).

**Table 3:** Cost-effectiveness of the TACScore strategy versus the WES strategy from the personal budget perspective

	Model 1: WES as a first-line test (USD)	Model 2: TACScore as a screening test (USD)
Assessments		
Outpatient appointment	1336.3	1336.3
Genetic consultation	676.2	64.4
Clinical consultation	1336.3	1336.3
*TBX6* Monogenic test		
Sanger sequencing	995.4	2061.9
ddPCR	1352.4	2801.4
Next generation sequencing		
WES	90868.4	49266.0
Other		
DNA extraction and shipping	2485.5	2485.5
Total cost	99050.5	59351.8
Total diagnostic time (d)	3656	2527
Total number of patients	83	83
Number of patients underwent WES	83	45
Total number of diagnoses	42	42
Average cost per patient (95% CI)	1193.4 (1185.5, 1201.3)	715.1 (594.5, 835.7)
Average cost difference (95% CI)	–	−478.3 (−599.1, −357.5)
Average diagnostic time per patient (95% CI) (d)	44.0 (43.2, 44.9)	30.4 (26.3, 34.6)
Average diagnostic time difference (95% CI) (d)	–	−13.6 (−17.8, −9.4)

*Abbreviations: WES* whole-exome sequencing, *TACScore TBX6*-associated congenital scoliosis risk score, *ddPCR* droplet digital polymerase chain reaction

### Bootstrapped simulations

**Fig. 2:**
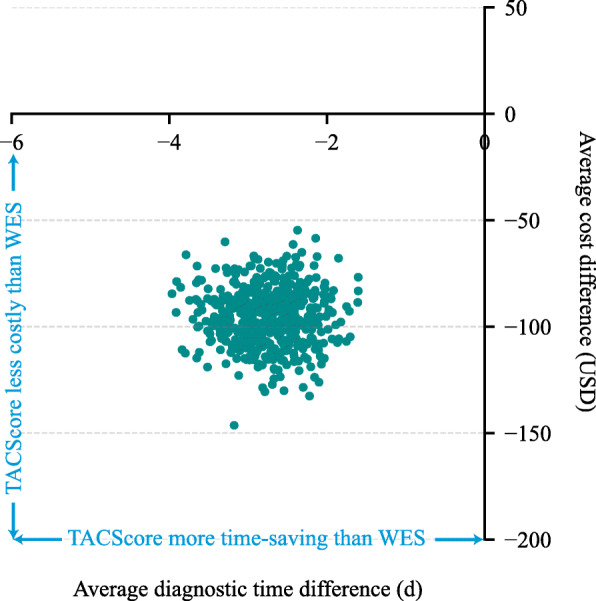
Cost-effectiveness plane for the WES strategy versus the TACScore strategy. The scatter plot contains 500 points, and each point represents the average cost differences and average diagnostic time differences of 500 bootstrapped simulations. Points below the x-axis and on the left side of the y-axis indicate that the TACScore strategy is less costly and more time-saving than WES. Therefore, for all of these simulations, the TACScore is considered cost-effective. Abbreviations: WES, whole-exome sequencing; TACScore, *TBX6*-associated congenital scoliosis risk score

To confirm our findings in a larger population and estimate the reliability of the calculations, we created 500 datasets. Each dataset consisted of 500 random samples from the study participants’ records. The cost and diagnostic time were calculated for each sample, and subsequently the average cost per patient and average diagnostic time per patient were calculated for each dataset. Thus, the average cost and diagnostic time difference were determined in each dataset. The cost-effectiveness plane demonstrated that the TACScore as a primary screening method was less costly and more time-saving than WES as the first-line test in 100% of the 500 simulations (Fig.2).

### Sensitivity analyses

To estimate the robustness of the cost-effectiveness analysis, we performed the one-way sensitivity analysis to examine how each variable affected the cost and diagnostic time. In terms of cost, the most influential variables were the costs of WES, Sanger sequencing, ddPCR and genetic consultation. The TACScore strategy remained cost-effective unless the cost of WES decreased to $8.8, or the costs of Sanger sequencing or ddPCR increased to $562.5 or $571.1, respectively. Regarding the diagnostic time, the TACScore strategy remained time-saving unless WES required less than 13d, or Sanger sequencing or ddPCR required more than 20d (Table 4).

**Table 4:** One-way sensitivity analysis for each variable in the cost-effectiveness analysis

Variables	Baseline Value	Range
Cost (USD)		
Outpatient appointment	16.1	Always
DNA extraction and shipping	29.9	Always
WES	1094.8	< 8.8
Sanger sequencing	23.7	> 562.5
ddPCR	32.2	> 571.1
Genetic consultation	16.1	Always
Clinical consultation	16.1	Always
Time (d)		
Outpatient appointment	1	Always
DNA extraction and shipping	1	Always
WES	37	< 13
Sanger sequencing	7	> 20
ddPCR	7	> 20
Genetic consultation	1	Always
Clinical consultation	1	Always

*Abbreviations: WES* whole-exome sequencing, *ddPCR* droplet digital polymerase chain reaction

**Fig. 3:**
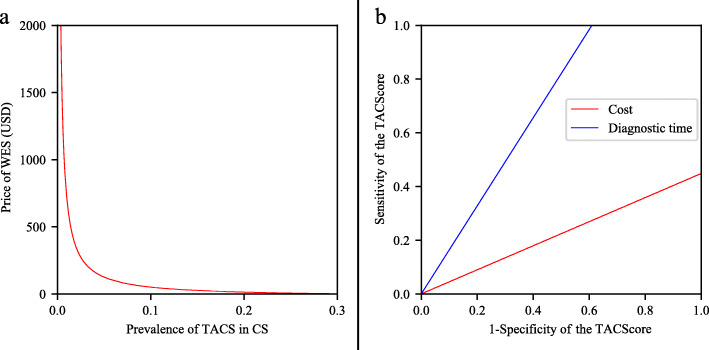
Two-way sensitivity analysis. **a** Two-way sensitivity analysis of the prevalence rate of TACS in CS versus the cost of WES. The x-axis shows the hypothesized prevalence of TACS in CS. The y-axis shows the threshold of the WES price. Above the red line, the TACScore is more cost-effective than the WES strategy. Under the red line, the WES strategy is more cost-effective than the TACScore strategy. **b** Two-way sensitivity analysis of the sensitivity of the TACScore versus the 1-specificity of the TACScore. The x-axis shows the hypothesized 1-specificity of the TACScore. The y-axis shows the hypothesized sensitivity of the TACScore. Above the red line, the TACScore strategy is less costly than the WES strategy. Above the blue line, the TACScore strategy is more time-saving than the WES strategy. Abbreviations: TACS, *TBX6*-associated congenital scoliosis; CS, congenital scoliosis; WES, whole-exome sequencing; TACScore, *TBX6*-associated congenital scoliosis risk score

The two-way sensitivity analysis of the prevalence of TACS in CS versus the price of WES revealed that the preferred strategy varied (Fig.3a). Specifically, above the red line, the TACScore strategy was more cost-effective than the WES strategy. The two-way sensitivity analysis of the 1-specificity of the TACScore versus the sensitivity of the TACScore was demonstrated in Fig.3b. In detail, above the red line, the TACScore strategy remained less costly than the WES strategy. Above the blue line, the TACScore strategy remained more time-saving.

## Discussion

The TACScore provides clinical geneticists with an efficient tool to evaluate the likelihood of a CS patient being diagnosed with TACS and, whether the patient is recommended to undergo a single gene or a genome-scale genetic test. Using the TACScore as the primary screening method counterfactually in the DISCO cohort, which provided a real and reliable molecular diagnostic yield of CS, improved the cost-effectiveness of the diagnostic process. Our study provides robust economic evidence for the translation from using the TACScore as a clinical assessment tool toward its application as a cost-effective screening test in molecularly diagnosing CS patients.

Delayed diagnosis and misdiagnosis are common for rare diseases and may cause serious and adverse effects on the patients [13–17]. Specifically, delayed diagnosis and misdiagnosis in the TACS lead to emotional distress, progression of scoliosis and delayed treatment. Besides, families whose child has a delayed diagnosis often consult multiple clinicians prior to receiving the correct molecular diagnosis [17]. An ill-defined genetic risk, frustration and disease progression brought by the diagnostic odyssey can cause anxiety and loss of reproductive confidence. In this condition, the patients’ families need to access the health system for treatment of anxiety and depression and pay additional medical costs. Thus, a prompt and accurate diagnosis can prevent disease progression, improve quality of life, and reduce financial burden. According to a recent study, lack of knowledge among health professionals, delays in obtaining test results and lack of access to appropriate tests are the leading causes of diagnostic delays [17]. Therefore, an accessible, fast and accurate screening test is needed for diagnosing TACS, which could be enabled by the TACScore. Evaluating patients’ phenotypes by the TACScore is both economical and time-saving, while WES is expensive, time-consuming and sometimes hard to access. The TACScore strategy successfully lowers the cost of diagnostic tests, and consequently more CS patients can receive a molecular diagnosis in time. Furthermore, a fast and accurate diagnosis provided by the TACScore can help the patients’ families alleviate the stress of disease progression, and enable them to end the diagnostic odyssey, and to restore reproductive confidence. In this case, using the TACScore as the first-line screening test has the capacity to improve CS patients’ prognosis by offering a prompt and correct molecular diagnosis.

A recent study found that a rare diseases cohort representing approximately 2.0% of an Australian state population accounted for between 4.6 and 10.5% of the state inpatient hospital costs, highlighting the marked disparity between the rare diseases population and their combined health-system costs [18, 19]. Interpretation of this result must take into consideration that a lack of knowledge and experience among health professionals lead to delayed and inaccurate diagnosis, followed by inappropriate referral pathways and expensive diagnostic procedures [10, 14, 19–22]. Specifically, CS impairs physical and mental abilities, shortens life expectancy and places a significant economic burden on the healthcare system [23]. With the inclusion of outpatient, mental health, and rehabilitation expenses, the total health-system cost attributed to CS is substantially greater than expected. Thus, to alleviate the significant financial burden of CS, we need an improved access to a cost-effective diagnostic test. As the TACScore is a simple and practical phenotype-based model, the health professionals can understand the characteristics of TACS readily and recognize its specific clinical phenotypes easily. Therefore, the health professionals can better identify TACS, order diagnostic tests and make an early diagnosis. Besides, as demonstrated by this study, using the TACScore as a screening test is less expensive than performing WES as the first-line test. Taken together, implementing the TACScore as a screening test can cut down the cost of CS patients to a level that is more proportionate to the prevalence of this rare condition.

This study also has some limitations. Although economic information, which supports the general use of the TACScore as screening test in molecularly diagnosing CS, was provided by this study, individual hospitals still need to evaluate the feasibility of using the TACScore in clinical practice. In China, WES is not in the scope of government medical insurance. Therefore, we conducted a cost-effectiveness analysis without considering different reimbursement rates. We admit that while the decision tree of the cost-effectiveness analysis varies from country to country, system to system, the TACScore system is still needed to be validated in other healthcare systems. This study collected the CS patients retrospectively, and designed the two models counterfactually, which merited further prospective studies to obtain high-level evidence.

## Conclusions

In summary, this retrospective study provides clinicians with economic evidence to integrate the TACScore into clinical practice. The TACScore can be considered a cost-effective tool when it serves as a screening test prior to performing WES.

## Methods

### Study design

This is a retrospective study to determine whether using the TACScore as a screening test for CS patients is more cost-effective than WES as the first-line test. We designed a cost-effectiveness analysis decision tree (Fig.1) using previously described techniques [24–27]. The diagnosis-related costs were analyzed to determine the differences of average cost and diagnostic duration between the two strategies.

### Patients collection

The DISCO cohort, which consisted of unrelated sporadic CS patients of Chinese Han descent, was recruited from Peking Union Medical College Hospital (PUMCH) between October 2010 and January 2018 [7]. Patients in DISCO cohort were retrospectively identified for possible inclusion in this study. The inclusion criteria were as follows: 1) patients who were diagnosed with CS; 2) patients who had available data regarding onset age, age of enrollment, sex and complete spinal images including X-ray, computed tomography (CT), and magnetic resonance imaging (MRI); 3) patients underwent WES for pursuing a molecular diagnosis; and 4) patients with informed consent for enrollment. The study was approved by the institutional review board of PUMCH.

### Cost-effectiveness analysis

We compared the differences of the cost and diagnostic duration under two counterfactual strategies (Fig.1):

- *The WES strategy, WES as the first-line test (counterfactual model 1)*. We modeled this counterfactual diagnostic pathway in which all recruited patients received WES as the first-line test.
- *The TACScore strategy, the TACScore as a screening test (counterfactual model 2).* Patients were evaluated by the TACScore first. A *TBX6* monogenic test was performed for patients who had TACScore was≥3. WES was implemented when the TACScore was ≤2.

For both of the strategies, the price of DNA extraction and shipping were calculated once, and each instance of a positive WES result required an appointment with a geneticist to interpret the report.

From the health care payer perspective, the overall expenditure and diagnostic time for all enrolled patients were calculated according to two diagnostic counterfactual strategies. From the personal budget perspective, the personal expenditure and diagnostic time for patients with a positive TACScore were calculated according to two diagnostic counterfactual strategies.

### WES and *TBX6* monogenic test

WES was performed in all patients as described previously [6, 28]. Sanger sequencing or/and ddPCR were performed to validate the causative variants.

*TBX6* monogenic test included Sanger sequencing of all exons and the approximately 1-kb upstream region of the *TBX6* gene and ddPCR testing of the 16p11.2 deletions [6]. For patients who underwent both Sanger sequencing and ddPCR, the one which was more time-consuming was regarded as the diagnostic time.

### Costs and diagnostic times

We obtained the costs and diagnostic times of the whole process of molecularly diagnosing a patient, which included outpatient appointment, DNA extraction and shipping, WES, Sanger sequencing, ddPCR, genetic consultation and clinical consultation (Table 1). The aforementioned data complied with the Chinese Medical Service Price Regulations. The total unit cost and diagnostic time of each patient were derived according to the decision tree (Fig.1). We converted Chinese Yuan (CNY) into American Dollars (USD) based on the exchange rate of CNY 1 = USD 0.161 on 30 June 2014 (source: http://fx.cmbchina.com). The duration of the diagnostic trajectory was calculated from the first clinical visit to when a genetic report was issued.

### Bootstrapped simulations and sensitivity analyses

We performed 500 bootstraps to estimate the reliability of the analysis. Each bootstrap represented 500 samples randomly collected from our cohort. We calculated the outcomes (including the average cost and the diagnostic time per patient) for each replicated data set, and then evaluated the distribution of each outcome.

We performed one-way and two-way sensitivity analyses to examine the robustness of the estimates. For the one-way sensitivity analysis, we determined the thresholds of the variables that alter the most cost-effective strategy according to the decision tree. For the two-way sensitivity analysis, we changed two variables simultaneously. Specifically, the two-way analysis was performed for two pairs of influential values (i.e. the prevalence of TACS in CS versus the price of WES, and 1-specificity of the TACScore versus sensitivity of the TACScore).

### Statistical analysis

We used the Student’s t-test for analyses of continuous variables. Statistical significance for differences was set as *P* = 0.05 (2-sided). The statistical analyses were performed using Python (version 3.7) and SPSS (version 21.0).

## Supplementary information

**Additional file 1.**

## Data Availability

The datasets used and/or analyzed during the current study are available from the corresponding author on reasonable request.

## Abbreviations

CS: congenital scoliosis
TACS: *TBX6*-associated congenital scoliosis
TACScore: *TBX6*-associated congenital scoliosis risk score
WES: whole-exome sequencing
DISCO: Deciphering disorders Involving Scoliosis and COmorbidities
ddPCR: droplet digital polymerase chain reaction
PUMCH: Peking Union Medical College Hospital
